# Alantolactone Improves Prolonged Exposure of Interleukin-6-Induced Skeletal Muscle Inflammation Associated Glucose Intolerance and Insulin Resistance

**DOI:** 10.3389/fphar.2017.00405

**Published:** 2017-06-29

**Authors:** Minjee Kim, Kwangho Song, Yeong Shik Kim

**Affiliations:** Natural Products Research Institute, College of Pharmacy, Seoul National UniversitySeoul, South Korea

**Keywords:** alantolactone, sesquiterpenoids, glucose intolerance, inflammation, insulin resistance, diabetes

## Abstract

The pro-inflammatory cytokine, Interleukin-6 (IL-6), has been proposed to be one of the mediators that link chronic inflammation to glucose intolerance and insulin resistance. Many studies have demonstrated the effects of IL-6 on insulin action in the skeletal muscle. However, few studies have investigated the effect of long-term treatment of IL-6, leading to glucose intolerance and insulin resistance. In the present study, we observed protective effects of alantolactone, a sesquiterpene lactone isolated from *Inula helenium* against glucose intolerance and insulin resistance induced by prolonged exposure of IL-6. Alantolactone has been reported to have anti-inflammatory and anti-cancer effects through IL-6-induced signal transducer and activator of transcription 3 (STAT3) signaling pathway. The relationship between IL-6 exposure and expression of toll-like receptor 4 (TLR4), involved in inflammation in the skeletal muscle, and the underlying mechanisms were investigated. We observed maximum dysregulation of glucose uptake after 40 ng/ml IL-6 induction for 24 h in L6 myotubes. Prolonged IL-6 exposure suppressed glucose uptake regulating alpha serine/threonine-protein kinase (AKT) phosphorylation; however, pretreatment with alantolactone activated AKT phosphorylation and improved glucose uptake. Alantolactone also attenuated IL-6-stimulated STAT3 phosphorylation, followed by an increase in expression of negative regulator suppressor of cytokine signaling 3 (SOCS3). Furthermore, IL-6-induced expression of pathogen recognition receptor, TLR4, was also suppressed by alantolactone pretreatment. Post-silencing of STAT3 using siRNA approach, IL-6-stimulated siRNA-STAT3 improved glucose uptake and suppressed TLR4 gene expression. Taken together, we propose that, as a STAT3 inhibitor, alantolactone, improves glucose regulation in the skeletal muscle by inhibiting IL-6-induced STAT3-SOCS3 signaling followed by inhibition of the TLR4 gene expression. Therefore, alantolactone can be a promising candidate for the treatment of inflammation-associated glucose intolerance and insulin resistance.

**GRAPHICAL ABSTRACT:**
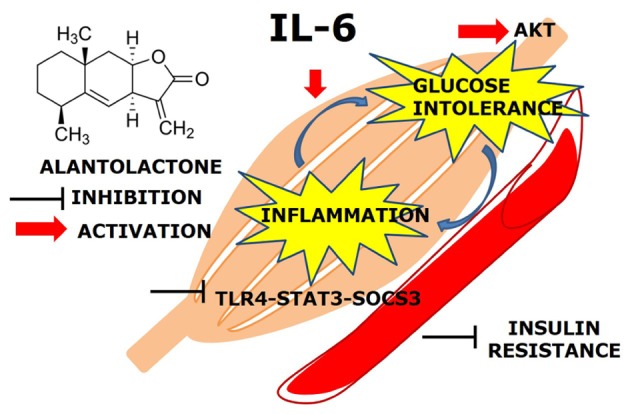
Effects of IL-6 in the skeletal muscle.

## Introduction

Skeletal muscle tissue accounts for over 80% of insulin-mediated glucose uptake and fatty acid oxidation (Breen et al., 2008). Skeletal muscle plays a fundamental role in glucose homeostasis through interactive cross-talk with hepatic and adipose tissue (Kim et al., 2008). Therefore, the decreased glucose transport in muscle tissue may lead to insulin resistance (Breen et al., 2008; Zygmunt et al., 2010).

Many studies have demonstrated insulin resistance in association with low-grade chronic inflammation (Kim and Sears, 2010; Chen et al., 2015). In the state of chronic inflammation, macrophage infiltration alters metabolic properties of muscle cells that produce inflammatory cytokines such as IL-6 and TNF-α (Kim et al., 2013). A recent study described the dual effects of IL-6 on insulin action in the skeletal muscle (Nieto-Vazquez et al., 2008). Specifically, short-term treatment with IL-6, improved glucose control and insulin sensitivity, whereas long-term treatment with IL-6 contributed to glucose intolerance and insulin resistance (Nieto-Vazquez et al., 2008; Kim et al., 2013). It is generally accepted that depletion of IL-6 improves glucose regulation and obesity in mouse model (Klover et al., 2005) and obesity-associated insulin resistance in type 2 diabetes in humans (Mashili et al., 2013).

In human skeletal muscle, it has been reported that long-term exposure of IL-6 downregulates expression of STAT3 and SOCS3 proteins (Senn et al., 2003; Kim et al., 2008; Mashili et al., 2013). STAT3 is implicated in development of IL-6-induced insulin resistance in cultured skeletal myotubes obtained from patients with impaired glucose tolerance (Kim et al., 2013). SOCS3 is associated with the IL-6–STAT3 pathway in insulin signaling (Senn et al., 2003) and has been reported to be increased in the skeletal muscle of severely obese or type 2 diabetes patients (Steinberg et al., 2006; Jorgensen et al., 2013). Herein, we tested the protein activation levels of pSTAT3 and SOCS3 in the skeletal muscle. Recent studies have suggested that SOCS3 blocks phosphorylation of IRS1 and downregulates PI3K complexes and phosphorylation of AKT (Ueki et al., 2004; Jorgensen et al., 2013). Therefore, we evaluated phosphorylation levels of AKT to validate glucose control in association with SOC3 expression.

Chronic inflammation partly impairs insulin action via toll-like receptor (TLR) activation, specifically, TLR2 and TLR4 (Kim and Sears, 2010). TLR4 is expressed in insulin target tissues, including the liver, adipose tissue, and skeletal muscle (Kim and Sears, 2010; Chen et al., 2015). Thus, activation of TLR4 may directly exacerbate insulin sensitivity, through activation of inflammatory kinases (Konner and Bruning, 2011). Previous data suggest that TLR signaling may also link chronic inflammation to insulin resistance in the skeletal muscle (Reyna et al., 2008; Kim and Sears, 2010; Konner and Bruning, 2011). Herein, we hypothesized that increased levels of IL-6 may be associated with increased levels of TLR4.

Alantolactone, a sesquiterpene lactone isolated from *Inula helenium* has been reported to have anti-inflammatory and anti-cancer properties (Chun et al., 2012, 2015). The mechanism underlying anti-inflammatory activity of alantolactone is inhibition of the STAT3 signaling pathway (Chun et al., 2012). Taking into account, its significant STAT3 inhibiting effect, we designed a study, wherein a glucose-intolerant and insulin-resistant state was induced using IL-6, which activates STAT3 phosphorylation in the skeletal muscle. Therefore, we hypothesized that alantolactone may exert anti-inflammatory effects in association with glucose intolerance. We observed improvement in IL-6-induced glucose intolerance by RNAi-mediated silencing of STAT3, suggesting that silencing of the STAT3 gene exerts positive effects on glucose homeostasis. We also examined the link between the IL-6–STAT3 pathways and TLR4 by siRNA and inhibitor studies.

Considering the importance of skeletal muscle in the regulation of glucose control and insulin resistance, alantolactone may be a potent candidate for the treatment of glucose intolerance and insulin resistant treatment in the future.

## Materials and Methods

### Reagents

All chemicals and reagents, unless specified, were purchased from Sigma–Aldrich (Sigma–Aldrich, MO, United States). Glucose uptake assay fluorescent 2-NBDG was purchased from Invitrogen (Carlsbad, CA, United States). IL-6 was purchased from Thermo fisher scientific (Waltham, MA, United States). Primary antibody pSTAT3, STAT3, pAKT, AKT, SOCS3, TLR4, and β-actin, as well as HRP-conjugated anti-rabbit and anti-mouse secondary antibodies were purchased from Santa Cruz (Dallas, TX, United States). Penicillin, streptomycin, DMEM (high glucose), fetal bovine serum (FBS) were obtained from GenDepot (Barker, TX, United States). siRNAs STAT3 and TLR4 were designed and created from Bioneer (Daejeon, South Korea). RNAiMAX for transfection was purchased from Invitrogen (Carlsbad, CA, United States).

### Plant Material and Preparation of Alantolactone

The root of *I. helenium* (Compositae), also known as elecampane, was purchased from the herb market in Jechun, Chungbuk of South Korea. Alantolactone was isolated from *I. helenium*, according to our recent paper (Kim et al., 2017). The purity was assessed over 98% by HPLC and the chemical structure was confirmed by ^1^H and ^13^C NMR (Supplementary Data).

### Cell Culture

L6 rat myotubes were obtained from the American Type Culture Collection (Manassas, VA, United States). L6 cells were grown in DMEM containing 10% FBS, 100 U/ml penicillin and 100 μg/ml streptomycin at 37°C in a 5% CO_2_ incubator. Differentiation was induced by 2% FBS medium. The experiments were performed after 6–7 days seeding.

### 2-NBDG Glucose Uptake Assay

L6 myoblasts were seeded and serum deprived for 24 h. Alantolactone was pre-treated for 4 h prior to IL-6 treatment for 24 h. Culture medium was removed and replaced with the culture medium with 500 μM florescent 2-NBDG (Molecular Probes-Invitrogen), a fluorescent derivative of glucose, for 3 h and stimulated with or without 100 nM insulin for 30 min. Supernatants were then removed and PBS buffer was added to each well. The fluorescent 2-NBDG images were determined by fluorescence microscopy (Olympus CKX41, ×200).

### Western Blotting

The protein samples (20 μg) were separated by SDS–PAGE, electro-blotted (BioRad) to the membrane, and blocked with skimmed milk for 1 h. The primary antibodies against pSTAT3, STAT3, pAKT, AKT, SOCS3, TLR4 and β-actin (Santa Cruz) were measured. All western blots were measured more than three times.

### siRNA Transfection

L6 cells were transfected with siRNA targeting STAT3 and TLR4 (Bioneer). The cells were seeded in the serum- and antibiotic-free media and transfected with siRNA-STAT3 and TLR4 (100 nM of each oligonucleotide sequence) or 50 nM scramble sequence according to the manufacturer’s instruction (RNAiMax, Invitrogen). After 72 h transfection, the cells were washed and treated with or without alantolactone for 4 h followed by 40 ng/ml IL-6 for 24 h. Protein was extracted with or without 100 nM insulin for 10–30 min for western blotting. The media containing 500 μM 2-NBDG (Invitrogen) was incubated for 3 h, followed by 30 min incubation with or without insulin for glucose uptake assay.

### Statistical Analyses

Comparisons were made via Student’s *t*-test or analysis of variance (ANOVA) followed by Dunnett’s test. All data are presented as the means ± standard error of mean (SE). *P*-values of <0.05 were considered as statistically significant.

## Results

### Alantolactone Improved Glucose Utilization after Prolonged Exposure of IL-6

**FIGURE 1:**
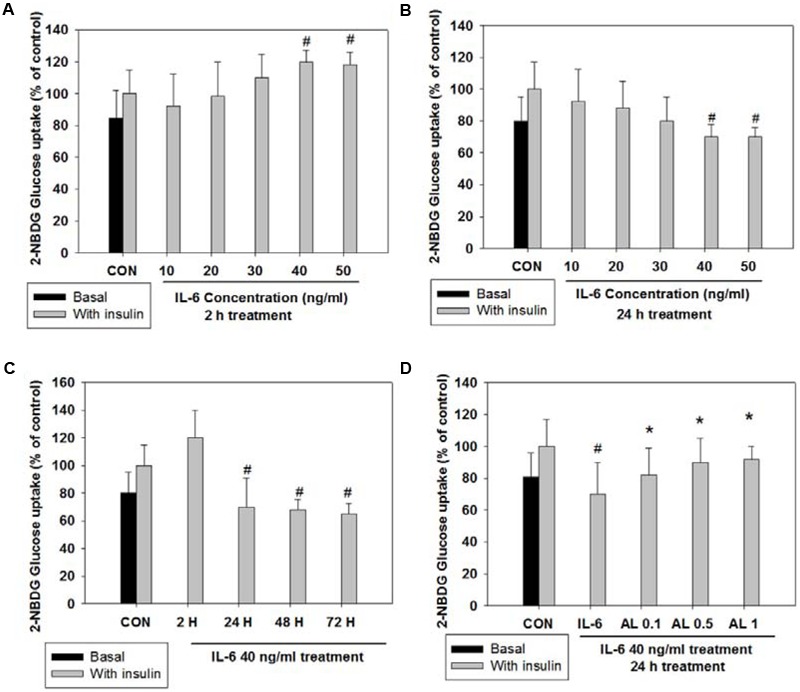
2-NBDG glucose uptake after acute (2 h) and chronic (24 h) IL-6-induced insulin-stimulated exposure in L6 skeletal muscle cells. Each value represents mean ± SD (standard deviation) from triplicate measurements (*n* = 3). **(A)** Acute IL-6 treatment in a different concentration (10–50 ng/ml) for 2 h. Insulin-stimulated glucose uptake peaked at 40 ng/ml (*n* = 3, ^#^*p* < 0.05 vs. insulin-stimulated control). **(B)** Chronic IL-6 treatment in a different concentration for 24 h. Insulin-stimulated glucose uptake significantly decreased from 40 ng/ml (*n* = 3, ^#^*p* < 0.05 vs. insulin-stimulated control). **(C)** IL-6 treatment (40 ng/ml) in different timeline (2, 24, 48, 72 h) with or without insulin 100 nM for 30 min. (*n* = 3, ^#^*p* < 0.05 vs. insulin-stimulated control). **(D)** Effects of alantolactone on 2-NBDG glucose uptake after chronic (24 h) IL-6 exposure. (*n* = 3, ^#^*p* < 0.05 vs. insulin-stimulated control, ^∗^*p* < 0.05 vs. insulin-stimulated IL-6).

**FIGURE 2:**
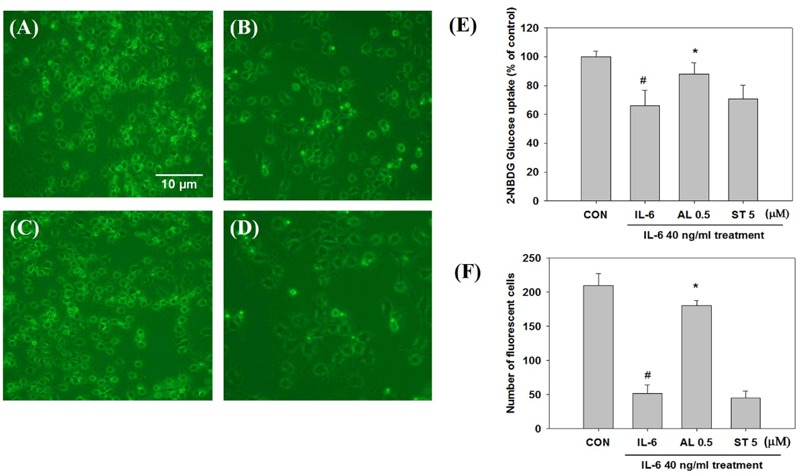
Glucose uptake observation by fluorescence microscopy after staining with 2-NBDG (×200 original magnification). Each value represents mean ± SD (standard deviation) from triplicate measurements (*n* = 3). **(A)** CON+insulin (control with insulin treatment); **(B)** IL-6+insulin (40 ng/ml IL-6 24 h exposure with insulin treatment) Scale bar: 10 μm; **(C)** AL 0.5+IL-6+insulin (0.5 μM alantolactone pretreatment for 4 h and IL-6 24 h exposure with insulin treatment); **(D)** ST 5+IL-6+insulin (5 μM Stattic pre-treatment for 4 h and IL-6 24 h exposure with insulin treatment); AL, alantolactone; ST, Stattic. **(E)** Effects of alantolactone and Stattic on 2-NBDG Glucose uptake level after IL-6 exposure (*n* = 3, ^#^*p* < 0.05 vs. insulin-stimulated control, ^∗^*p* < 0.05 vs. insulin-stimulated IL-6). **(F)** Number of fluorescent cells after 2-NBDG glucose uptake quantified by using an image J program.

To validate the impact of acute (2 h) and chronic (24 h) IL-6 treatment on glucose homeostasis, we examined insulin-stimulated 2-NBDG uptake. Insulin-stimulated glucose uptake peaked at a concentration of 40 ng/ml after 2 h of IL-6 treatment; after 24 h of IL-6 treatment, glucose uptake decreased in a dose-dependent manner showing a glucose-intolerant state (**Figures 1A,B**). The concentration of 40 ng/ml IL-6 was chosen for further study. To confirm that glucose intolerance is induced by prolonged IL-6 exposure, we evaluated glucose uptake after IL-6 treatment for 2, 24, 48, and 72 h. The level of glucose uptake increased 2 h after treatment, but decreased 24 h onward (**Figure 1C**). Then, cells were pretreated with alantolactone for 4 h in order to evaluate its protective effect against prolonged IL-6 treatment. Alantolactone reversed IL-6-induced insulin-stimulated glucose uptake level to that of the control group, indicating its protective effect against IL-6 treatment (**Figure 1D**). The level of glucose uptake was determined by using fluorescence microscopy (×200 original magnification) after staining with 2-NBDG (**Figure 2**). Stattic, a well-known STAT3 inhibitor (5 μM), was used as another control. The fluorescent intensity was the most significant in the control group, and diminished after IL-6 exposure (**Figures 2A,B**). These changes were significantly reversed by 0.5 μM alantolactone pre-treatment (**Figure 2C**), but no significant change was observed with Stattic pretreatment (**Figure 2D**). The glucose uptake level was also measured by using a microplate reader. Alantolactone improved glucose uptake after IL-6 exposure, but in case of Stattic, it remained unchanged, which corresponds to the previous result (**Figure 2E**). The number of fluorescent cells were evaluated using the image J program. The level of fluorescence significantly diminished by IL-6 exposure, and increased under pretreatment with alantolactone, showing a similar trend as in glucose uptake assay. This result suggests a protective effect of alantolactone against IL-6 exposure (**Figure 2F**).

### Alantolactone Reversed IL-6-Induced Insulin-Stimulated STAT3 Phosphorylation and SOCS3 Expression

**FIGURE 3:**
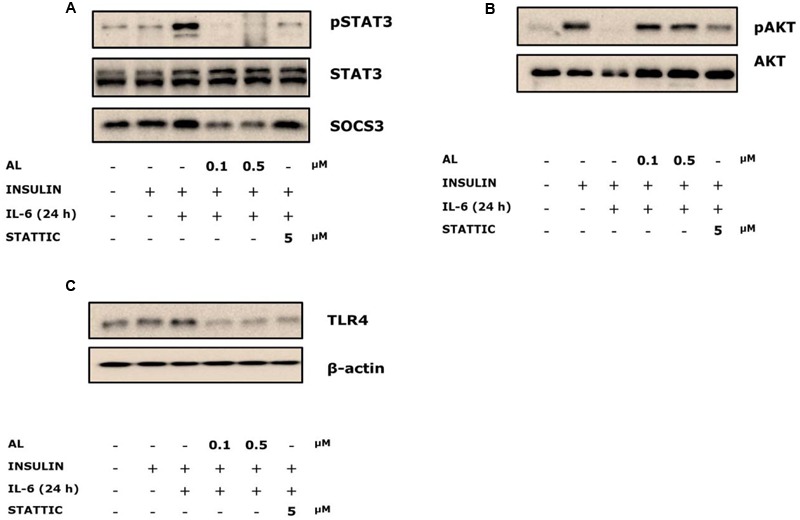
Western blots of alantolactone and Stattic in IL-6-induced insulin-stimulated L6 cells. All western blots were measured more than three times. **(A)** Effects of alantolactone and Stattic on IL-6-induced insulin-stimulation on STAT3 phosphorylation and SOCS3 activation. **(B)** Effects of alantolactone and Stattic on IL-6-induced insulin-stimulated AKT phosphorylation. **(C)** Effects of alantolactone and Stattic on IL-6-induced insulin-stimulated TLR4 gene expression.

Cells were pretreated with alantolactone and Stattic for 4 h, and then stimulated with 40 ng/ml IL-6 for 24 h. STAT3 phosphorylation significantly increased after IL-6 exposure, and both alantolactone and Stattic pretreatment reversed these changes. SOCS3 links the IL-6–STAT3 pathway to insulin signaling and plays a critical role in the development of insulin resistance in type 2 diabetes (Kim et al., 2013). SOCS3 is known to be downstream of STAT3; hence, it was investigated in association with STAT3. Expression of SOCS3 increased after IL-6 exposure. However, this increase was attenuated by alantolactone pretreatment (**Figure 3A**). Pretreatment with Stattic resulted in reduction of STAT3 phosphorylation as expected, but no change was observed in SOCS3 expression, indicating selective inhibitory activity of Stattic in the skeletal muscle.

### Alantolactone Activated IL-6-Induced Insulin-Stimulated AKT Phosphorylation

To examine glucose uptake regulation, AKT phosphorylation was evaluated. IL-6 treatment suppressed AKT phosphorylation, whereas alantolactone reversed the suppression to the control level (**Figure 3B**). However, pretreatment with Stattic did not show any significant change compared to IL-6-stimulated group, which supports the previous glucose uptake result.

### Alantolactone Reversed IL-6-Induced Insulin-Stimulated TLR4 Gene Expression

Toll-like receptor 4 expression was evaluated in association with chronic IL-6 treatment. We demonstrated increased expression levels of TLR4 gene in the IL-6-treated insulin-stimulated group. Both alantolactone and Stattic suppressed these expressions (**Figure 3C**).

### siRNA-Based Gene Silencing of STAT3 Improved Glucose Uptake

**FIGURE 4:**
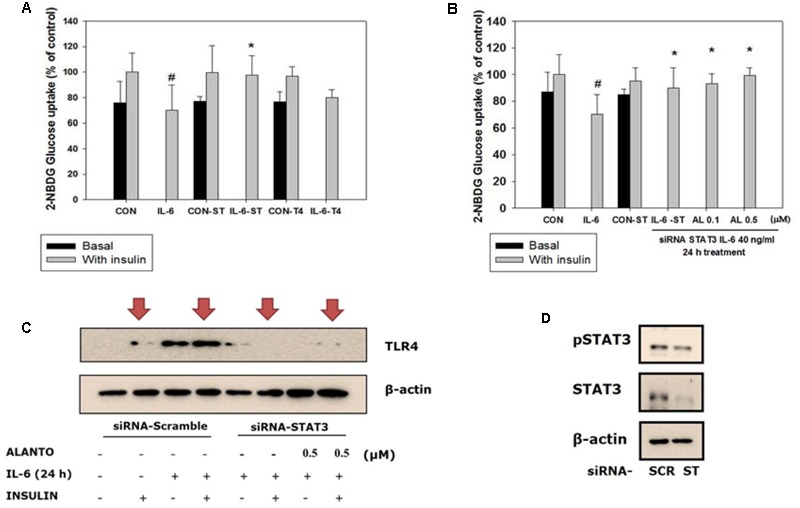
Silencing of STAT3. Each value represents mean ± SD (standard deviation) from triplicate measurements (*n* = 3). **(A)** L6 transfection with siRNA-STAT3 for 72 h followed by 40 ng/ml IL-6 for 24 h (*n* = 3, ^∗^*p* < 0.05 vs. insulin-stimulated IL-6-SC); CON-SC, control-scrambled-siRNA; IL-6-SC, IL-6 treated-scrambled siRNA; CON-ST, control-siRNA STAT3; IL-6-ST, IL-6 treated-siRNA STAT3; CON-T4, control-siRNA TLR4; IL-6-T4, IL-6 treated-siRNA TLR4. **(B)** 2-NBDG glucose uptake of siRNA-STAT3 and siRNA-TLR4. SiRNA-STAT3 reversed IL-6 induced insulin-stimulated glucose uptake level to the control (*n* = 3, ^∗^*p* < 0.05 vs. insulin-stimulated IL-6-SC); A, alantolactone. **(C)** Western blot of IL-6 induced insulin-stimulated scrambled-siRNA on TLR4 gene expression (*n* = 3). **(D)** Western blot of scrambled-siRNA and siRNA-STAT3. ^#^*p* < 0.05 vs. insulin-stimulated control.

To examine the role of STAT3 and TLR4 in the development of glucose intolerance, we tested IL-6-induced insulin-stimulated uptake of 2-NBDG in the skeletal muscle (**Figure 4A**). After 72 h of transfection with siRNA-STAT3, followed by 24 h of IL-6 treatment, the glucose level in the insulin-stimulated siRNA-STAT3 was reversed to that of the IL-6 non-treated scrambled siRNA group (**Figure 4B**). Compared to siRNA-TLR4, transfection with siRNA-STAT3 showed a more improved glucose uptake. Therefore, it was chosen to study further signaling pathway.

### TLR4 Gene Expression Diminished in Myotubes Treated with siRNA-STAT3

To evaluate IL-6-induced insulin-stimulated expression of the TLR4 gene via STAT3 in L6 myotubes, we used treatment with siRNA-STAT3 to examine the association between TLR4 and STAT3. After siRNA-STAT3 transfection, pretreatment with alantolactone for 4 h was followed by IL-6 treatment for another 24 h. Expression of the TLR4 gene was diminished in myotubes treated with siRNA-STAT3. The same trend was observed and after alantolactone pretreatment (**Figures 4C,D**).

## Discussion

**FIGURE 5:**
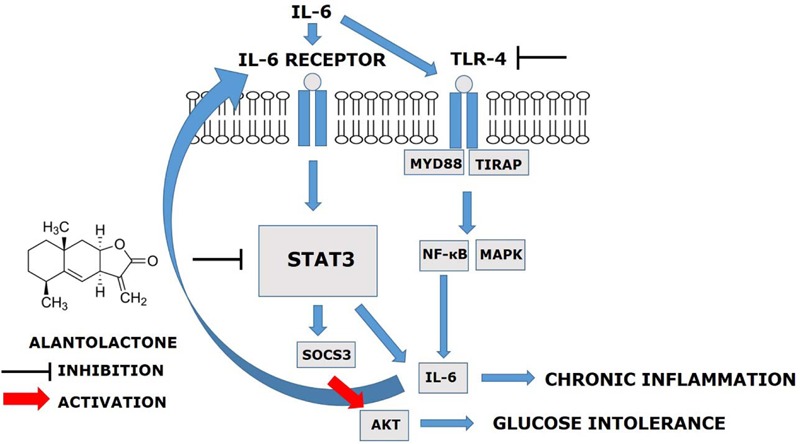
Alantolactone pathway scheme. Alantolactone suppresses IL-6-stimulated TLR4 expression via STAT3 phosphorylation and SOCS3 expression, which activates AKT phosphorylation.

The main finding of this study was that, prolonged (24 h) exposure of IL-6 mediates TLR4 gene expression via STAT3-SOCS3 activation and induces glucose intolerance and inflammation in the skeletal muscle. Alantolactone pretreatment showed a protective effect against chronic IL-6 treatment and increased glucose uptake level, suggesting its potential activity on glucose intolerance and insulin resistance (**Figure 5**).

Elevated IL-6 levels in the plasma and tissues are generally accepted as negative regulation in metabolism (Kim et al., 2013). In diabetic patients, IL-6 level was reported to be elevated by more than 25 times compared to that in the normal individuals (Goyal et al., 2012). STAT3, which is downstream of IL-6 signaling, is reported to be associated with IL-6-induced insulin resistance and gluconeogenesis in the liver and muscle (Kim et al., 2013). In the present study, we discovered that prolonged IL-6 stimulation increased TLR4 expression. We also used siRNA to determine the mechanisms and the crosstalk between IL-6 and TLR4 expression via STAT3 in the development of insulin resistance and glucose intolerance in the skeletal muscle. After STAT3 silencing, the level of increased TLR4 gene expression diminished, supporting the hypothesis of STAT3’s role as a mediator between IL-6 and TLR4. Previous reports suggested that obese and type 2 diabetes patients have increased the level of TLR4 expression and involvement of NF-κB in human myotubes and p-STAT3 protein activation (Reyna et al., 2008). We observed that both alantolactone and Stattic attenuated TLR4 expression, indicating the protective effect of alantolactone against muscle inflammation mediated through the IL-6–STAT3–TLR4 pathway. Recent studies in the liver and skeletal muscle have shown that SOCS3 over-expression affects proximal insulin signaling, by blocking IRS1, PI3K, and AKT phosphorylation (IRS-1–PI3K–AKT pathway) (Ueki et al., 2004; Kim et al., 2008; Jorgensen et al., 2013). In the obese mouse model, SOCS3 was genetically deleted from the skeletal muscle, and this resulted in enhanced glucose regulation and improved insulin sensitivity (Jorgensen et al., 2013). These data cohere with our results in that, chronic IL-6 exposure activated SOCS3 expression. Alantolactone pretreatment suppressed SOC3 expression, but in case of Stattic, it remained unchanged. We then measured AKT phosphorylation, which is associated with glucose regulation and found decreased phosphorylation after IL-6 stimulation. However, alantolactone pretreatment activated AKT phosphorylation, supporting positive effects of alantolactone in improving glucose uptake through SOCS3-AKT signaling. Interestingly, AKT phosphorylation was not changed by Stattic pretreatment, indicating that Stattic selectively inhibits STAT3 phosphorylation and does not affect glucose regulation. This result supports SOCS3 involvement in insulin signaling, which results in improving glucose intolerance in the skeletal muscle.

Accumulating clinical evidence suggest that monocytes/macrophages play a critical role in the pathogenesis of insulin resistance by infiltrating insulin target tissues (Reyna et al., 2008; Chen et al., 2015). Cytokines such as TNF-α and IL-6 secreted by multiple tissues, are recognized as the inflammatory mediators that cause insulin resistance by reducing the expression of glucose transporter4 (GLUT4) and IRS-1 (Chen et al., 2015). These effects are reported to exert JAK–STAT signaling pathway activation followed by SOCS3 expression (Chen et al., 2015). IL-6 is also reported to induce insulin resistance by blocking PI3K and AKT pathway and impair glycogen synthesis by downregulating microRNA200s and upregulating friend of GATA 2 (FOG-2) (Chen et al., 2015). Recent study (Chun et al., 2015) reported alantolactone’s inhibitory effect on inducible and constitutively activated STAT3, nuclear translocation suppression, and the DNA binding activity of STAT3 *in vitro*. This result supports our hypothesis that the anti-inflammatory effect of alantolactone may have suppressed chronic inflammation by inhibiting STAT3 activation, followed by TLR4 expression induced by IL-6 exposure.

## Conclusion

These results of this study indicate that alantolactone exerts its anti-inflammatory effects by inhibiting IL-6-induced insulin-stimulated glucose intolerance and insulin resistance in the skeletal muscle. To our knowledge, this study is the first to report that alantolactone suppresses IL-6-stimulated TLR4 expression via STAT3 phosphorylation and SOCS3 activation. Therefore, alantolactone may have a great potential for the treatment of chronic inflammation-associated metabolic disorders, such as insulin resistance and type 2 diabetes.

## Author Contributions

Cell culture experiments, 2-NBDG glucose uptake, siRNA cell transfection and western blots were completed by MK. Isolation of alantolactone was completed and provided by KS. Study protocol approval and manuscript correction were conducted by YK as a corresponding author.

## Conflict of Interest Statement

The authors declare that the research was conducted in the absence of any commercial or financial relationships that could be construed as a potential conflict of interest. The reviewer MH and handling Editor declared their shared affiliation, and the handling Editor states that the process nevertheless met the standards of a fair and objective review.

## Abbreviations

2-NBDG: 2-(*N*-(7-nitrobenz-2-oxa-1,3-diazol-4-yl)amino)-2-deoxyglucose
AKT: alpha serine/threonine-protein kinase
GLUT4: glucose transporter 4
IL-6: Interleukin-6
IRS-1: insulin receptor substrate-1
PI3K: phosphoinositide 3-kinase
SOCS3: suppressor of cytokine signaling 3
STAT3: signal transducer and activator of transcription 3
TLR4: toll-like receptor 4
TNF-α: tumor necrosis factor alpha
