# Supplementary material for: Alantolactone Improves Prolonged Exposure of Interleukin-6-Induced Skeletal Muscle Inflammation Associated Glucose Intolerance and Insulin Resistance
Source: Front Pharmacol. 2017 Jun 29;8:405. doi: 10.3389/fphar.2017.00405 (PMC5489625; doi:10.3389/fphar.2017.00405)
Supplement: Supplementary file 1 [file Table_1.DOCX]

**Supplementary data Table 1.** ^1^H and ^13^C NMR data of alantolactone (*δ* in ppm, *J* in Hz, 500 and 125 MHz in CDCl_3_).

| No. | ^1^H | ^13^C |
| --- | --- | --- |
| 1 | 1.38-1.45 (2H, m) | 41.5 |
| 2 | 1.49-1.60 (2H, m) | 16.4 |
| 3 | 1.73-1.83 (2H, m) | 32.4 |
| 4 | 2.43 (1H, m) | 37.3 |
| 5 |  | 139.5 |
| 6 | 5.13 (1H, d, *J*= 3.8) | 118.5 |
| 7 | 3.56 (1H, m) | 39.1 |
| 8 | 4.80 (1H, m) | 76.1 |
| 9 | 1.53 (1H, dd, *J*_1_= 15.0, *J*_2_= 2.5)  2.09 (1H, dd, *J*_1_= 15.0, *J*_2_= 2.5) | 42.3 |
| 10 |  | 32.3 |
| 11 |  | 148.8 |
| 12 |  | 170.1 |
| 13 | 5.60 (1H, d,  *J*= 1.5)  6.18 (1H, d,  *J*= 1.8) | 121.3 |
| 14 | 1.18 (3H, s) | 28.3 |
| 15 | 1.07 (3H, d, *J*= 7.5) | 22.3 |

**
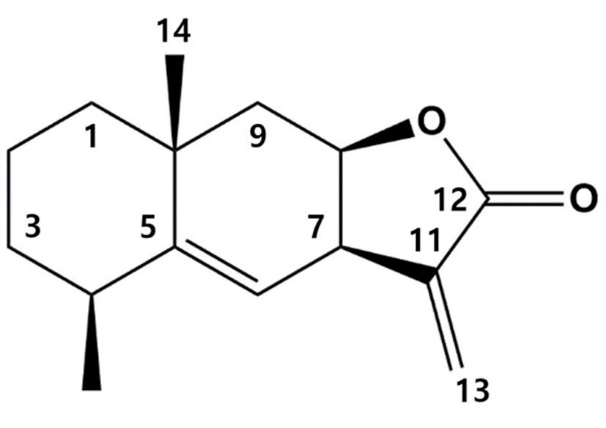
**
